# R-848 triggers the expression of TLR7/8 and suppresses HIV replication in monocytes

**DOI:** 10.1186/1471-2334-12-5

**Published:** 2012-01-14

**Authors:** Hua Nian, Wen-Qing Geng, Hua-Lu Cui, Ming-jia Bao, Zi-ning Zhang, Min Zhang, Ying Pan, Qing-Hai Hu, Hong Shang

**Affiliations:** 1 Key Laboratory of AIDS Immunology of Ministry of Health, Department of Laboratory Medicine, No.1 Hospital of China Medical University, No.155 Nanjing North Street, Heping District, Shenyang 110001, China; 2 Center for Disease Control and Prevention, Jiamusi, Heilongjiang 154007, China

**Keywords:** Toll-like receptor, HIV, Monocytes, R-848

## Abstract

**Background:**

Toll-like receptors (TLR) 7 and 8 are important in single-stranded viral RNA recognition and may play a role in HIV infection and disease progression. We analyzed TLR7/8 expression and signaling in monocytes from HIV-infected and uninfected subjects to investigate a pathway with new potential for the suppression of HIV replication.

**Methods:**

Eighty-one HIV-infected and uninfected subjects from Liaoning and Henan provinces in China participated in this study. Monocytes were isolated from subjects' peripheral blood mononuclear cells by magnetic bead selection. TLR7 and TLR8 mRNA was measured using quantitative real-time reverse transcriptase PCR. R-848 (resiquimod) was used as a ligand for TLR7 and TLR8 in order to 1) assess TLR7/8-mediated monocyte responsiveness as indicated by IL-12 p40 and TNF-α secretion and 2) to examine HIV replication in cultured monocytes in the presence of R-848.

**Results:**

We found that expression of TLR7/8 mRNA in peripheral blood monocytes decreased with disease progression. TLR7 expression was decreased with stimulation with the TLR7/8 agonist, R-848, in vitro, whereas TLR8 expression was unaffected. Following R-848 stimulation, monocytes from HIV-infected subjects produced significantly less TNF-α than those from uninfected subjects, but trended towards greater production of IL-12 than stimulated monocytes from uninfected subjects. R-848 stimulation also suppressed HIV replication in cultured monocytes.

**Conclusions:**

Our study provides evidence that the TLR7 and TLR8 triggering can suppress HIV replication in monocytes and lead to postpone HIV disease progression, thereby offering novel targets for immunomodulatory therapy.

## Background

Infection with HIV-1, the causative agent of AIDS, is characterized clinically by a long asymptomatic period of latency preceding the development of AIDS. Even during this period of clinical latency, the virus replicates continuously and causes new rounds of infection. In recent years, many researchers have focused on adaptive immune responses against HIV infection. To date, the mechanisms that modulate HIV replication during this clinically latent stage are not completely clear and studies of HIV immunotherapy and vaccination have not shown great progress.

The importance of innate immunity in HIV infection is becoming increasingly apparent [1-3]. Innate immunity serves as the first line of defense against microbial pathogens and is also responsible for the initiation of inflammatory responses through the release of a variety of cytokines, chemokines, and antimicrobial factors. Monocytes, the precursors of macrophages and dendritic cells, are involved in the innate immune response via cognate interactions and production of proinflammatory cytokines, such as interferons (IFNs), IL-12, IL-6 and tumor necrosis factor alpha (TNF-α). In particular, toll-like receptors (TLRs) that are expressed on monocytes can signal cytokine release, cellular activation, and up-regulation of the MHC Class I or Class II [4], and thus help link the innate and the adaptive immune responses.

TLRs are a family of pattern recognition receptors that mediate essential mechanisms of innate immunity against microbial pathogens [5-7]. TLRs are grouped by their preferences for conserved structural motifs of microorganisms. TLR3, 7, 8, and 9 are implicated in anti-viral defense [5,8]. A recent study reported that TLR7/8 can recognize uridine-rich ssRNA (ssRNA40) derived from the HIV-1 long terminal repeat (LTR) [9], suggesting that TLR7/8 may be involved in HIV infection as other investigators have previously reported [10,11].

It is still unknown whether TLR7/8 expression in monocytes is related to disease progression in HIV-infected patients or whether it represents a protective factor in those patients who are slow disease-progressors. Both TLR7 and 8 recognize single-stranded viral RNA (ssRNA) [12,13], lead to NF-κB activation [14], and promote the production of cytokines such as IL-12 and TNF-α [15]. NF-κB is critical for the transcription of most immune response genes, inducing both types I and II cytokines [16]. Ironically, there are NF-κB binding sites located within the HIV LTR [14] that can bind NF-κB and enhance HIV replication. Thus, the role of TLR7/8 signaling in monocytes' response to HIV replication merits further investigation.

In this study, we examined monocyte expression of TLR7 and 8 mRNA from subjects with HIV over the course of disease progression. We also assessed TLR7/8-dependent cytokine secretion and HIV replication in monocytes stimulated with the synthetic TLR7/8 ligand R-848. We demonstrate that HIV disease progression is influenced by TLR7/8 expression and signaling in monocytes, offering additional targets in the pursuit of treatments and cures for AIDS.

## Methods

### Study subjects

Sixty-three HIV-1-infected subjects from Liaoning and Henan provinces in China were enrolled in the study. All had been previously diagnosed using an anti-HIV antibody screening test (Vironostika, Organon Tednika, The Netherlands) which was confirmed by immunoblot-based testing (Gene Lab Diagnostics, Singapore). HIV-infected subjects were classified into three clinical groups. Subjects in the first group were characterized as "slow progressors" (SPs), with persistent CD4^+ ^cell counts greater than 500 cells/μl, no anti-retroviral therapy, and no clinical signs of disease for at least 10 years. Subjects in the second group were characterized as "chronic HIV infection subjects", who had CD4^+ ^cell counts between 200 and 500 cells/μl with no antiretroviral therapy and no AIDS-defining symptoms. Patients in the third group were labeled "AIDS subjects", consisting of patients with < 200 CD4^+ ^cells/μl and/or the appearance of opportunistic infections or AIDS-defining symptoms according to the World Health Organization classification. According to these criteria, the study included 20 SPs, 25 chronic HIV infection subjects, 18 AIDS subjects and 18 HIV-negative healthy persons (Table 1). All control subjects had normal blood cell counts, normal levels of hemoglobin, and normal liver function and did not have any history of immune system disease. Blood samples from subjects were obtained when opportunistic infection was not present. Samples from HIV-infected and control subjects were always handled in parallel. All subjects included here gave informed consent and written approval was obtained from the appropriate hospital ethics committees. All investigations were conducted according to Helsinki Declaration guidelines.

**Table 1:** Details of study subjects in each group

Study group	Females/Males (age)^a^	CD4 Count (cells/μl)^b^	Plasma Viral Load (copies/ml)^b^	HCV Positive n^c^	HBV Positive n^c^	Receiving HAART therapy n^c^
SPs(20)	8/12 (47: 33-56)	637 (563-722)	47500 (24500-407750	5	0	0

HIV chronic(25)	11/14 (44: 30-54)	411 (328-487)	11000 (2005-75000)	5	1	0

AIDS(18)	7/11 (48: 37-59)	107 (55-151)	4100 (790-16000)	4	1	4

Control(18)	9/9 (46: 36-54)	823 (524-1135)	ND^d^	0	0	ND^d^

^a^, Numbers of Females/Numbers of Males (median age in years: age interquartile range)

^b^, Median (interquartile range)

^c^, n, Positive Numbers

^d^, ND, not data

### Isolation, culture, and stimulation of monocytes

Peripheral blood mononuclear cells (PBMCs) were isolated from buffy coats of heparinized blood by Ficoll-Hypaque (Amersham Biosciences) density gradient centrifugation; monocytes were further isolated using CD14^+^-conjugated magnetic beads (Miltenyi Biotech, Germany) for the positive selection according to the manufacturer's instructions. The purity of the monocyte population was > 97% as determined by flow cytometry with phycoerythrin (PE)-conjugated anti-CD14 antibody (BD Pharmingen). Cell viability of monocytes at isolation was > 90% as determined by trypan blue. Monocytes were cultured in RPMI-1640 supplemented with 10% fetal bovine serum, 2 mM L-glutamine, 25 mM HEPES, 100 IU/ml penicillin G and 100 μg/ml streptomycin, and were cultured at 5 × 10^5 ^cells/0.5 ml/well on 48-well flat bottom tissue culture plates following the protocol described by Bekeredjian-Ding et al. [15]. The antibiotics were added to prevent bacterial growth; cultures grown in media lacking antibiotics did not display different TLR7/8 expression. Monocytes were stimulated with the TLR7/8 agonist R-848 (resiquimod, Alexis) at 2.5 μg/ml [15] or medium alone (controls) in cultures at 37°C, 5% CO_2 _for 24 h, at which point monocyte cytokine secretion was fully functional and the levels of TLR 7/8 expression were also evaluated. Cell-free supernatants were harvested after a 24-h culturing period and the concentrations of IL-12p40 (the bioactive form of IL-12) and TNF-α were determined using an ELISA assay (R&D and eBioscience, respectively). All supplemented media were found free of endotoxin (lack of TNF-α induction in monocytes) [17]. HIV RNA load in supernatant was also measured to assess viral replication before and after culture of monocytes from HIV-infected patients. Monocytes were washed three times after separation and before culture; HIV RNA was not detected in any initial monocyte culture. Monocytes were cultured in 48 well plates with approximately 5 × 10^5 ^cells/0.5 ml/well which was sufficient to produce enough virus in 48 h to exceed detection limits [18].

### HIV RNA load measurement

HIV-1 RNA levels in culture supernatants were extracted from each sample with the Nuclisens extractor (bioMérieux) and quantified using the Nuclisens EasyQ (version 1.1) assay (bioMérieux) following the manufacturer's instructions.

### Determination of CD4^+ ^cell counts

CD4^+ ^cell counts were measured using a FACS Calibur flow cytometer (Becton-Dickinson). A single-platform lyse/no-wash procedure was performed using Trucount tubes and TriTEST CD4-FITC/CD8-PE/CD3-PerCP reagents (BD Biosciences).

### Measurement of TLR7/8 mRNA expression

Total RNA was extracted from monocytes using the RNeasy Protect Mini Kit (QIAGEN) according to the manufacturer's protocol. The concentration of total RNA extracted from monocytes was determined by measuring optical density at 260/280 nm. Reverse transcriptase polymerase chain reaction was carried out using a kit from the Takara according to the manufacturer's protocol. The RT reaction consisted of 500 ng of total RNA, 0.5 μl Oligo dT (50 μmol), 0.5 μl random hexamers (100 μmol), 2 μl 5 × PrimeScript Buffer, adjusted to 10 μl with Rnase-Free dH_2_O. Reaction conditions were set at 37°C for 15 min, 85°C for 5 s, and 4°C until finished. The resulting cDNA was stored at -20°C for subsequent polymerase chain reaction (PCR) amplification. Expression analysis was performed using quantitative real-time PCR with SYBR Green I (TakaRa, Japan) on an ABI 7500 real time PCR system (Applied Biosystem Inc., USA). Primers for TLR7 and TLR8 were designed using Primer Express program (ABI) and their specificity was examined with BLAST queries in the National Center for Biotechnology Information (NCBI) database. The following 5' to 3' oligoucleotides were used: For TLR7, AATGTCACAGCCGTCCCTAC (sense) and GCGCATCAAAAGCATTTACA (antisense); for TLR8, TGTGATGGTGGTGCTTCAAT (sense) and ATGCCCCAGAGGCTATTTCT (antisense). Thermal cycler parameters included 40 cycles at 95°C (5 s) and 60°C (34 s). The dissolution curve conditions were 95°C for 15 s, 60°C for 1 min, and 95°C for 15 s. All gene-specific mRNA expression values were normalized against the housekeeping gene, glyceraldehyde 3-phosphate dehydrogenase (GAPDH). After amplification, melting curves were generated automatically by the ABI 7500; only those showing a single high production peak were considered to be valid amplifications. PCR amplification of the RNA sample alone (without reverse transcriptase treatment) was performed as a control; no products of the TLR7/8 gene were detected indicating that there was no relevant DNA contamination of the RNA sample.

### Statistical analysis

Data are depicted as means ± SEM or given as median values (IQRs). All statistical tests used for data analysis were performed using the SPSS Version 11.5 software package (SPSS, Inc. Chicago). Differences among the groups were compared using the Fisher's least significant difference test (LSD) and correlation was determined using the Pearson test for unpaired and normal data. Differences between groups were compared using the Mann-Whitney *U *test, and correlation was analyzed using the Spearman rank test for unpaired and non-normal data. Wilcoxon signed rank tests were used for comparisons of non-normal paired data. Probability values were two-sided and considered to be significant when *p *< 0.05.

## Results

### Expression of toll-like receptor 7/8 in peripheral blood monocytes is associated with AIDS progression

**Figure 1:**
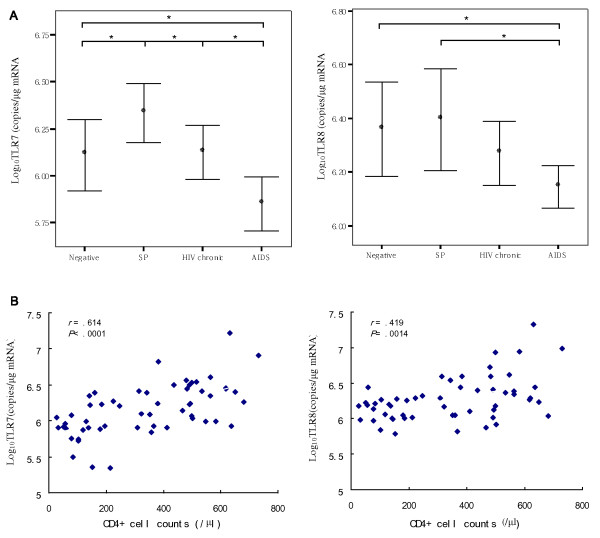
**Expression of Toll-like receptor 7/8 in peripheral blood monocytes is associated with AIDS progression and correlates positively with CD4^+ ^cell counts**. (**A**) Expression of TLR7 was significantly increased in slow progressors (SPs) versus uninfected subjects, chronic HIV infected subjects, and subjects with AIDS. TLR7 expression was also significantly increased in subjects with chronic HIV infection versus AIDS. TLR7 in subjects with AIDS was significantly decreased compared to uninfected subjects. The expression of TLR8 was significantly decreased in subjects with AIDS compared with both uninfected subjects and SPs,*p < 0.05. Comparisons were made with one-way ANOVA LSD tests. All data are expressed as the mean ± SEM values from experiments performed in triplicate. (**B**) The expression of TLR7 and TLR8 was significantly correlated with CD4^+ ^cell counts. Data was analyzed using the Spearman rank correlation with a level of significance of α = 0.05.

To better understand the effect of HIV infection on the expression of TLR7/8 in monocytes, total RNA was extracted from purified monocytes of HIV-infected subjects and uninfected subjects; TLR7 and TLR8 mRNA was then measured using real-time PCR. Figure 1A shows that TLR7 mRNA levels in monocytes were significantly increased in SPs versus uninfected subjects, chronic HIV infection subjects, and AIDS subjects (*p *= 0.0483, *p *= 0.0473, and *p *< 0.0001 respectively), but were significantly decreased in AIDS subjects in comparison to uninfected subjects (*p *= 0.0252) and chronic HIV infection subjects (*p *= 0.0108). The expression of TLR8 was significantly decreased in subjects with AIDS compared with both uninfected subjects and SPs (*p *= 0.0414, *p *= 0.0169). No difference in TLR8 was seen among SPs, chronic HIV infection subjects, and uninfected controls. In addition, we found that TLR7 and TLR8 mRNA levels are significantly correlated with CD4^+ ^cell counts (Figure 1B) (TLR7, r = 0.614, *p *< 0.0001; TLR8, r = 0.419, *p *= 0.0014). Taken together, expression of TLR7 and TLR8 in monocytes was correlated with AIDS disease progression and appeared to decrease as with advancing severity of disease from SPs to AIDS.

### R-848 influence on toll-like receptor 7/8 expression in monocytes in vitro

**Figure 2:**
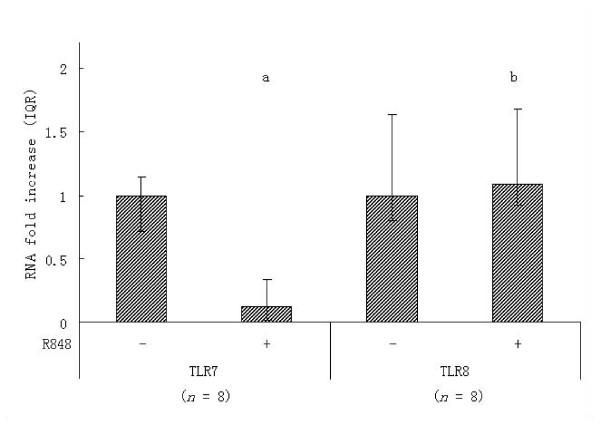
**R-848 alters Toll-like receptor expression in vitro**. Freshly isolated monocytes from uninfected (*n *= 3) and HIV-infected (*n *= 5) subjects were cultured in the presence of R-848 or media alone for 24 h. TLR7/8 mRNA then quantified using real-time PCR. R-848 stimulation significantly decreased expression of TLR7 in monocytes (^a^*p *= 0.025) but did not alter TLR8 expression (^b^*p *= 0.944). Data was analyzed using the Wilcoxon signed ranks test. Bars indicate the interquartile range (IQR).

To determine if innate immune signals may play a role in the decrease of TLR expression in monocytes in HIV infection, we evaluated the effect of the TLR7/8 ligand, R-848 (resiquimod) [19], on TLR7 and TLR8 expression in cultured monocytes. R-848 functions as an analogue of retroviral ssRNA to initiate signaling through both TLR7 and TLR8. Monocytes from five HIV-infected and three uninfected subjects were tested ex vivo (Table 2). Stimulation with R-848 significantly decreased expression of TLR7 in monocytes derived from both HIV-infected and uninfected subjects(*p *= 0.025). Treatment with R-848 did not alter TLR8 expression (*p *= 0.944) (Figure 2). Expression of TLR7/8 in monocytes from either HIV-infected or uninfected subjects appears the same (data not shown).

**Table 2:** Details of HIV infected subjects from whom monocytes were isolated and cultured ex vivo

Subject No.^a^	Sex (age)^c^	CD4 Count (cells/μl)	CD8 Count (cells/μl)	Plasma Viral Load (copies/ml)	HCV^b^	HBV^b^	HIV Subtype
1	M(42)	332	1326	18800	P	P	CRF07-BC

2	F(38)	412	1373	92300	N	N	CRF01-AE

3	M(45)	368	463	71000	P	N	B

4	M(22)	484	1333	60100	N	N	B

5	M(40)	364	842	3200	N	N	CRF01-AE

6	M(46)	265	421	140000	N	N	CRF01-AE

7	M(33)	328	485	63000	N	N	CRF01-AE

8	F(45)	480	737	< 10	P	N	B'

9	F(45)	359	1064	3600	N	P	B'

10	M(32)	276	869	830000	P	P	B'

^a ^Monocytes from subjects no.2,3,4,5,7 were used to analyze R-848 influence on expression of TLR7/8; Monocytes from subjects no.1-10 were used to examine TLR7/8 signaling capacity; monocytes from subjects no.1-9 were used to examine HIV replication suppression mediated by TLR7/8. No subjects received HAART therapy

^b ^Minor or no biochemical signs of ongoing liver inflammation (alanine aminotransferase level < 70); P, Positive; N, Negative

^c ^M, male; F, female; age in years

### Response of TLR7 and TLR8 in monocytes contributes to HIV infection

**Figure 3:**
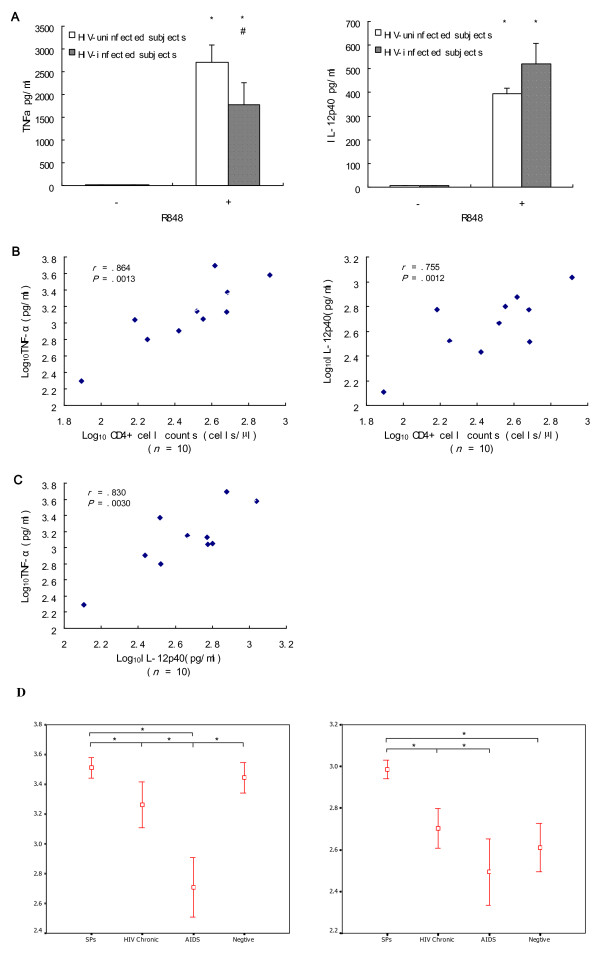
**Proinflammatory cytokine secretion mediated by TLR7/8 in monocytes varied based on HIV-infection status and correlated positively with CD4^+ ^cell counts of peripheral blood in HIV-infected subjects**. Ex vivo monocytes from uninfected (n = 10) and HIV-infected (n = 10) subjects were cultured in the presence of TLR 7/8 ligand R-848 or media alone for 24 h. Culture supernatants were assessed for cytokine levels using an ELISA assay. (**A**) R-848 stimulation produced significantly greater TNF-α production in monocytes of both HIV-infected and uninfected subjects compared with unstimulated samples (*p *< 0.0001); stimulated uninfected subjects produced more TNF-α than stimulated HIV-infected subjects (*p *= 0.0412). R-848 also induced significantly greater IL-12p40 production in monocytes of both HIV-infected (*p *< 0.0001) and uninfected subjects compared with unstimulated monocytes (*p *< 0.0001). No statistically significant increase in IL-12p40 was observed in HIV-infected versus uninfected subjects (*P *= 0.198). TNF-α levels were analyzed using the Mann-Whitney U two-tailed test. Independent *t *test were used to analyze IL-12p40 levels. The significant differences are represented as p value (* *p *< 0.0001 compared with unstimulated by R-848, # *p *= 0.0412 compared with uninfected subjects). All data are expressed as the mean ± SEM values from experiments performed in triplicate. (**B**) Levels of TNF-α and IL-12p40 secreted by monocytes via TLR7/8 were significantly correlated with CD4^+ ^cell counts in the ten HIV-infected individuals. (**C**) TNF-α exhibited a significant positive correlation with IL-12p40 in HIV-infected individuals. Data was analyzed using a Pearson correlation. Experiments were repeated at least 2 times with similar results. (**D**) The level of TNF-α and IL-12p40 secretions of triggered monocytes derived from 31 subjects belonging to the four categories (7 subjects of SPs, 10 of HIV Chronic, 7 of AIDS and 7 of Negative) were described (*p < 0.05).

To assess whether alterations in TLR7 and TLR8 expression associated with HIV infection resulted in altered TLR signaling, we compared the induction of TNF-α and IL-12p40 by R-848 in monocytes from ten chronic HIV infection subjects (Table 2) and nine uninfected subjects. Stimulation of monocytes with R-848 resulted in the significant production of TNF-α and IL-12p40 compared with no production in media alone (Figure 3A). Significantly lower TNF-α production was seen in HIV-infected subjects compared with uninfected subjects. The production of IL-12p40 trended towards elevated expression but this increase was not significant. Additionally, we analyzed the relationship between TLR7 or TLR8 expression and production of these two cytokines but found no correlation (data not shown). Lack of correlation suggests that increased TLR7/8 expression does not result in increased cytokine secretion. Therefore, we propose that increased TLR7/8 expression may enhance the cell's ability to recognize ssRNA of virions. Further studies are warranted to confirm our observations.

Interestingly, we found that the production of TNF-α and IL-12p40 secreted by monocytes via TLR7/8 was significantly correlated with CD4^+ ^cell counts in HIV-infected individuals (Figure 3B). This data indicates that CD4^+ ^cell counts could reflect TLR7/8-mediated monocyte secretion function. So we observed the level of TNF-α and IL-12p40 secretion of monocytes from subjects of four groups in order to learn monocytes responsiveness during the HIV infection progression. We have found that TNF-α and IL-12p40 secretions of triggered monocytes were significant difference at each HIV infection disease stage (Figure 3D, *p < 0.05).

### R-848 inhibits HIV replication in monocytes through the TLR7/8 pathway

**Figure 4:**
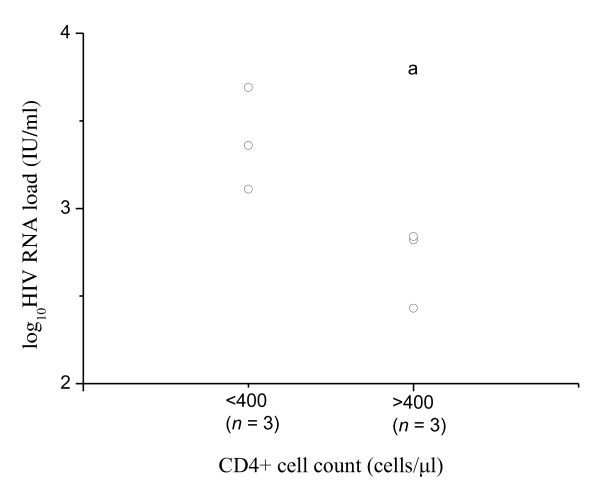
**HIV RNA levels in monocyte culture supernatants are associated with peripheral blood CD4^+ ^cell counts**. Monocytes isolated from HIV-infected subjects (*n *= 9) were cultured in vitro. Supernatants in six subjects produced low levels of productive viral particles. HIV-infected subjects were divided into two groups by CD4^+ ^cell count around a median of 400 cells/μl. HIV RNA levels (viral load, logarithmically scaled) of each group are shown. The group with < 400 CD4^+ ^cells/μl had higher HIV replication levels than the group of > 400 CD4^+ ^cells/μl (^a^*p *= 0.0323). Data was analyzed using an independent *t *test.

The correlation of monocyte cytokine secretion capacity with an individual's HIV disease progression status may imply a protective role for the TLR7/8 pathway. We investigated whether the TLR7/8 pathway could contribute to HIV replication in monocytes, since studies have shown that monocytes are considered underestimated HIV-1 viral reservoirs [20]. We first isolated monocytes from nine chronic HIV-infected subjects (Table 2) and cultured them for 48 h to assess the ability of HIV to replicate in monocytes. HIV RNA loads in the culture supernatants were found to be low in six samples and were not detected in the other three. We found that culture supernatants of monocytes from subjects with CD4^+ ^cell counts < 400 cells/μl had significantly higher HIV RNA loads than those with > 400 cells/μl (Figure 4).

**Figure 5:**
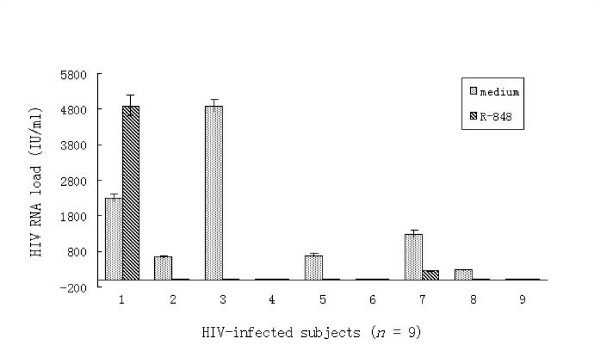
**HIV RNA levels in culture supernatants of monocytes were partially suppressed by R-848 via the TLR7/8 pathway**. Monocytes isolated from chronic HIV-infected subjects (*n *= 9) were cultured in vitro, of which six cases produced detectable low-level viral loads in the culture supernatant. Viral loads of five cases significantly decreased in presence of R-848 (*p *= 0.0431) and increased in one case. The other three cases had no detectable HIV replication with or without R-848 stimulation. Data was analyzed using the Wilcoxon signed-rank test were used with a level of significance of α = 0.05. All data are expressed as the mean ± SEM from experiments performed in triplicate.

R-848 has been found to be a TLR7/8 agonist with demonstrated antiviral and anti-tumor activity [21,22]. Oral administration of R-848 has previously been tested against chronic HCV infection [23]. Importantly, R-848 has been shown to block HIV replication in acutely infected PBMCs by triggering TLR7/8 [24]. To further understand whether the TLR7/8 triggering could impact HIV replication in monocytes specifically, we cultured monocytes from subjects with chronic HIV infections with R-848 for 48 h and monitored supernatant HIV RNA load as an indicator of HIV replication. We found that HIV replication in culture supernatants significantly decreased in five cases, increased in one case, and showed no replication with or without R-848 in the other three cases (Figure 5). Cytokine levels and TLR mRNA were quantified in the culture supernatant exhibiting increased HIV replication; TNF measured 1399 pg/ml, IL-12 measured 464 pg/ml, TLR 7 mRNA was 5.87 copies/μg, and TLR8 mRNA was 6.13 copies/μg (Subject #1 in Table 2). Subject #1's TLR functional status showed no distinguishing feature but his culture was infected with HIV subtype (CRF07-BC). Whether this particular HIV subtype may be able to escape TLR 7/8's effect on replication merits further investigation.

## Discussion

This study, to our knowledge, represents the first analysis of the expression of TLR7/8 on monocytes during HIV disease progression and the role of TLR7/8 triggering in monocytes on viral replication.

Monocytes are vital members of the innate immune system as they are precursors to professional antigen presenting cells (APCs). However, monocytes have been implicated as a viral reservoir during HIV infection based on the detection and recovery of replication-competent virus from circulating monocytes isolated from HIV-positive individuals [20,25,26]. In our study, we isolated blood monocytes from nine HIV-infected subjects and cultured the samples in vitro. Monocytes from six of nine HIV-infected subjects exhibited HIV replication, which indicates that peripheral blood monocytes of HIV-infected patients contain replication-competent HIV particles.

Recently, Almodovar et al. [27] reported that peripheral blood monocytes do not seem to be latent sources of HIV in the presence of suppressive HAART (highly active retroviral therapy); however, in the absence of suppressive HAART, monocytes may become infected with HIV. In our study, although all nine patients were HAART-naïve, three did not show HIV replication which suggests that inhibition of HIV replication may be host-mediated [28,29]. However, we cannot rule out that this result could be explained by our 48-h culture protocol, which may have been insufficient to allow detectable viral replication in these three samples.

Several studies have demonstrated that, even in individuals with viral loads suppressed below detection for prolonged periods of time, HIV-1 still continues to replicate at very low levels in monocytes and that infected monocytes can transmit HIV-1 to other susceptible cells [20,26,30]. Suppression or elimination of HIV in infected subjects' monocytes would be an important method of preventing rebound infection. In our study, we provide evidence that TLR7/8 triggering by the TLR7/8 agonist, R-848, can suppress HIV replication in monocytes. Moreover, a TLR7/8-initiated pathway can induce a protective adaptive immune response that leads to suppression of the pathogen [31-33], which indicates that there are multiple roles for TLR7/8 in an antiviral response.

The mechanism of HIV-1 latency in monocytes is not fully understood. Given that the TLR7/8 receptors on monocytes can recognize viral ssRNA and thus mediate an antiviral immune response, we considered whether the replication of HIV could be inhibited by triggering the TLR7/8 signaling pathway. Our data demonstrate that R-848 can inhibit HIV replication in monocytes cultured from HIV-infected subjects, which suggests that this inhibition is related to the TLR7/8 signaling pathway and is dependent on monocytes themselves and not on input from other cell types.

We also investigated whether inhibition of replication could be related to the role of proinflammatory factors secreted by monocytes after agonist stimulation. Several studies have reported that TNF-α may induce HIV replication in vitro [34,35]. Other studies have noted a general increased in circulating TNF-α during HIV infection [36,37] We compared HIV-infected and healthy subjects by examining TNF-α and IL-12p40 in monocyte culture supernatants. We observed reduced TNF-α secretion by monocytes from HIV-infected patients following stimulation with R-848. In this respect, our results differ from previous results that show increased monocyte TNF-α secretion due to stimulation with gp120 and HIV virions [38-40]. As R-848 has a molecular structure similar to ssRNA, it is ideal for studying TLR7/8 signaling. However, R-848 cannot substitute for treatment with gp120 or complete HIV virions. Other components of the HIV virion may provide a significant source of stimulus for innate immune activation [41].

Among the six patients with positive HIV replication, we found that patients with lower CD4^+ ^cell counts had higher HIV replication levels, which supports the findings of Innocenti et al. [42]. The higher HIV replication levels indicated that the monocytes contained high levels of HIV DNA as compared to monocytes from subjects with high CD4^+ ^cell counts (which is an established indicator of host immune status). Thus, we hypothesized that TLR7/8 expression may vary HIV infection stage.

Indeed, we found that TLR7 and TLR8 expression levels in monocytes declined as a function of the severity of HIV infection (i.e. slow progression to chronic HIV infection to AIDS). TLR7 expression was decreased significantly at each subsequent stage of HIV infection, while TLR8 expression decreased significantly from baseline levels only at the AIDS stage. Different levels of TLR7/8 expression in different stages of HIV infection suggest that their role in monocyte function also varies according to HIV infection stage. To test this difference further, we used the TLR7/8 ligand R-848 [19] to activate the TLR7/8 signaling pathway [43] in vitro. Our findings show that TNF-α and IL-12p40 secretions were decreased significantly at each subsequent HIV infection stage, which indicated that the decreased TLR7 expression of monocytes should be linked to a lower response to R-848. Meanwhile, our findings also show that TLR7 expression levels are decreased significantly after monocytes are stimulated by R-848 in vitro, which corresponds to the observed pattern of TLR7 expression levels in monocytes across disease progression stages.

This data suggests that TLR7 is more responsive and hypersensitized to its ligands, while TLR8 expression in monocytes in vitro was stable after stimulation with R-848, which corresponds to the conserved in vivo TLR8 expression in monocytes of subjects from the SPs and HIV chronic stages. Hence this study reveals differences in TLR7 and TLR8 expression in monocytes from HIV-infected subjects both in vivo and after in vitro stimulation; however, further studies are needed to elucidate a mechanistic explanation of altered TLR expression in HIV-infected monocytes.

TLR 7/8 may recognize HIV ssRNA. Binding of the appropriate ligands results in the recruitment of the adaptor protein Myeloid Differentiation Factor 88 (MyD88)followed by various IL-1 receptor-associated kinase (IRAK) family members. TNF receptor-associated factor 6 (TRAF6) is also recruited and finally the NF-κB and mitogen-activated protein kinases (MAPKs) are activated. These events result in the induction of inflammatory cytokines and chemokines [44]. Thus, TLR 7 binding to its ligand could result in the production of cytokines; decreased TLR 7 expression would theoretically lead to a drop in cytokine secretion. However, our study found no correlation between TLR expression and TNF or IL-12 secretion (data not shown). We speculate that the expression level of TLR 7 may influence the frequency with which ligands are recognized; the higher the level of TLR expression, the greater chance that a ligand such as ssHIV RNA will be detected.

Our study demonstrates that TLR 7/8 activation elicits an antiviral response in primary monocytes with increased TLR 7 expression at the initial disease (SP stage); thus, we can speculate that at the time of initial HIV-infection, HIV ssRNA may be recognized by TLR 7 and activate signaling pathways resulting in MyD88 activation and subsequent production of proinflammatory cytokines such as IFNs which could increase TLR 7/8 expression [45]. Persistent immune activation in HIV is thought to contribute to pathogenesis by progressively disturbing cytokine expression, functional organization of the immune system [46], and decreasing TLR expression (AIDS stage). Further studies are needed to examine this possibility. Interestingly, our observation of decreased TLR7 and TLR8 mRNA expression in monocytes differs from the increase in TLR7 and TLR8 mRNA expression as Lester et al. reported [47] in PBMCs, which suggests that monocytes experience a unique change in TLR7/8 expression distinct from other PBMCs.

Our study is preliminary and since it is performed in restricted conditions its extrapolation to clinical applications is challenging. It has several limitations. First, the number of cases is limited and this should be taken into consideration when interpreting the results. Nevertheless, to our knowledge, no other study has reported that R-848 inhibited HIV replication specifically in monocytes nor have other studies documented the association between TLR7/8 expression in monocytes and HIV disease progression. Second, TLR7/8 expression was only measured at the mRNA level; analysis of protein levels will increase our understanding of the phenomenon. However, Song et al. [10] have confirmed that enhanced expression of TLR7 mRNA in CD8^+ ^T cells corresponds to increased TLR7 protein expression. Finally, we have not yet undertaken sophisticated mechanistic studies of the variations in TLR7/8 signaling pathway which may occur during progressive stages of HIV infection.

## Conclusions

Our study reveals a relationship between TLR7/8 mRNA expression levels in monocytes and HIV disease progression. Furthermore, our data indicate that HIV replication can be suppressed via TLR7/8 ligation in vitro. Further analysis of the TLR7 and TLR8 pathway may contribute to the understanding of the immunopathogenesis of HIV infection and may ultimately offer novel targets for immunomodulatory therapy.

## Competing interests

The authors declare that they have no competing interests.

## Authors' contributions

HS and WQG conducted the experiments. HN, HLC, YP and MJB carried out all the test. MJB, QHH and MZ participated in the statistical analysis of the study. HS and ZNZ provided a critical review of the manuscript. HS designed the study and supervised the laboratory procedures and the data analysis. HN analyzed and interpreted the results and wrote the manuscript. All authors contributed to writing the manuscript and approved its final version.

## Pre-publication history

The pre-publication history for this paper can be accessed here:

http://www.biomedcentral.com/1471-2334/12/5/prepub
